# Normothermic machine perfusion attenuates hepatic ischaemia‐reperfusion injury by inhibiting CIRP‐mediated oxidative stress and mitochondrial fission

**DOI:** 10.1111/jcmm.17062

**Published:** 2021-11-16

**Authors:** Wenyan Liu, Yang Fan, Hongfan Ding, Dan Han, Yang Yan, Rongqian Wu, Yi Lv, Xinglong Zheng

**Affiliations:** ^1^ National Local Joint Engineering Research Center for Precision Surgery & Regenerative Medicine First Affiliated Hospital of Xi’an Jiaotong University Xi’an China; ^2^ Department of Blood Purification First Affiliated Hospital of Xi’an Jiaotong University Xi’an China; ^3^ Xi'an Medical University Xi’an China; ^4^ Department of Cardiovascular Surgery First Affiliated Hospital of Xi’an Jiaotong University Xi’an China

**Keywords:** CIRP, ischaemia‐reperfusion injury, mitochondrial fission, oxidative stress

## Abstract

Extracellular cold‐inducible RNA‐binding protein (CIRP) is a proinflammatory mediator that aggravates ischaemia‐reperfusion injury (IRI). Normothermic machine perfusion (NMP) could effectively alleviate the IRI of the liver, but the underlying mechanism remains to be explored. We show that human DCD livers secreted a large amount of CIRP during static cold storage (CS), which is released into the circulation after reperfusion. The expression of CIRP was related to postoperative IL‐6 levels and liver function. In a rat model, the CIRP expression was upregulated during warm ischaemia and cold storage. Then, rat DCD livers were preserved using CS, hypothermic oxygenated machine perfusion (HOPE) and NMP. C23, a CIRP inhibitor, was administrated in the HOPE group. Compared with CS, NMP significantly inhibited CIRP expression and decreased oxidative stress by downregulating NADPH oxidase and upregulating UCP2. NMP markedly inhibited the mitochondrial fission‐related proteins Drp‐1 and Fis‐1. Further, NMP increased the mitochondrial biogenesis‐related protein, TFAM. NMP significantly reduced inflammatory reactions and apoptosis after reperfusion, and NMP‐preserved liver tissue had higher bile secretion and ICG metabolism compared to the CS group. Moreover, C23 administration attenuated IRI in the HOPE group. Additionally, HL‐7702 cells were stimulated with rhCIRP and C23. High rhCIRP levels increased oxidative stress and apoptosis. In summary, NMP attenuates the IRI of DCD liver by inhibiting CIRP‐mediated oxidative stress and mitochondrial fission.

## 1 INTRODUCTION

Numerous clinical studies have confirmed that normothermic machine perfusion (NMP) can effectively reduce ischaemia‐reperfusion injury (IRI) and promote the recovery of liver function after liver transplantation.^1^, ^2^, ^3^ However, the molecular mechanism describing how NMP attenuates IRI in the donation after cardiac death (DCD) liver remains unclear. Cold‐inducible RNA‐binding protein (CIRP) is expressed at low levels in various tissues and can be induced by stress such as hypothermia and hypoxia.^4^, ^5^ Recently, growing evidence shows that extracellular CIRP can function as a novel damage‐associated molecular pattern (DAMP) molecule that triggers proinflammatory responses.^6^, ^7^, ^8^ In addition, blocking CIRP protein significantly increases the survival rate in a mouse hepatic ischaemia‐reperfusion model.^9^ The DCD liver experiences hypotension before donation and suffers from hypothermia and hypoxia during cold storage (CS). These factors cause the liver to secrete a large amount of CIRP, which mediates IRI after transplantation.

Mitochondrial dysfunction and excessive reactive oxygen species (ROS) are among the main mechanisms of hepatic IRI.^10^ The mitochondria are the main target of ROS damage and the main site of ROS production. In hepatic ischaemia‐reperfusion (I/R), elevated ROS directly causes oxidative damage to mitochondria and activates the apoptosis pathway, which aggravates tissue injury.^11^ In addition, upregulating the expression of dynamin‐related protein 1 (Drp‐1) and fission 1 (Fis‐1) increases mitochondrial fission in hepatic I/R. Excessive mitochondrial fission leads to mitochondrial fragmentation and ROS generation.^12^, ^13^ Li et al.^14^ found that CIRP released from damaged tissue induces NADPH oxidase‐derived ROS via TLR‐4/MyD88 signalling to promote fragmentation of mitochondrial DNA.

Normothermic machine perfusion maintains physiological conditions by the supplementation of key metabolic substrates, namely oxygen and adenosine triphosphate (ATP), during liver perfusion.^10^ Further, NMP could inhibit CIRP secretion by avoiding hypothermia and hypoxia, thereby offering an opportunity for organ reconditioning and rehabilitation. In the present study, we hypothesized that a large amount of CIRP is released from the DCD liver, which induces NADPH oxidase‐derived ROS and leads to mitochondrial dysfunction. However, NMP may inhibit CIRP‐mediated oxidative stress and mitochondrial fission, thereby reducing liver IRI.

## 2 MATERIALS AND METHODS

### 2.1 Clinical specimens and patient data

This study was approved by the Ethics Committee of the First Affiliated Hospital of Xi'an Jiaotong University. Informed consent was obtained from all patients prior to the study. A total of 41 specimens of cold preservation solution [the University of Wisconsin solution (UW)] were randomly collected from patients undergoing liver transplantation in the Department of Hepatobiliary Surgery from April 2015 to September 2016. During this period, serum samples from another 30 patients were collected at the following five time points, immediately after anaesthesia, 5 min after reperfusion, 1 h after reperfusion, 2 h after reperfusion, and 1 day after surgery. The specimens were stored at −80℃ until further analysis. In addition, the CS time of DCD livers and the serum transaminase levels on the first day after transplantation were recorded.

### 2.2 Rat DCD model

Male Sprague‐Dawley rats (9–10 weeks old) were used in this study. Animals were maintained with free access to a standard laboratory diet and water. All animal protocols were approved by the Animals Care and Use Committee of Xi'an Jiaotong University. The animals were fasted for 12 h before surgery. The rats were anaesthetized with isoflurane and injected with 2‐ml heparin saline (250 U/ml) via the dorsal penile vein. The abdominal cavity was opened with a cross‐abdominal incision. An intravenous catheter (20G) was inserted into the portal vein and another intravenous catheter (24G) was inserted into the hepatic artery after ligating and dividing the communication branches between the spleen and stomach, and the splenic artery. In addition, the common bile duct was intubated. Cardiac arrest was induced by an incision in the diaphragm leading to hypoxia and was recorded as the beginning of warm ischaemia. The livers were subjected to different warm ischaemia treatments for 0, 10 and 30 min (WI 0, WI 10, and WI 30, *n* = 6/group). After that, heparin saline (50 U/L) was infused via the hepatic artery using a peristaltic pump (Longer BQ50‐1J) at a speed of 2 ml/min for 20 min. The inferior vena cava was cut to provide an outlet. Then, the liver was collected for further preservation.

### 2.3 Preservation of DCD livers

Three liver preservation strategies were used for DCD livers (WI 30): static cold storage (CS), hypothermic oxygenated machine perfusion (HOPE), and NMP. For the CS group (*n* = 6), the DCD livers were perfused with 70 ml UW solution through the portal vein cannula. Then, the livers were immersed in UW solution and maintained at 4℃ for 4 h. For the HOPE group (*n* = 6), the perfusate was maintained at 4℃ and perfused through the portal vein cannula at a speed of 8 ml/min for 4 h. The perfusate consisted of 70 ml UW solution, 250 U heparin, 20,000 U penicillin and 2 mg hydrocortisone, with the pO_2_ maintained at 300–400 mmHg using a hollow fibre oxygenator (Xijian Medical). The NMP system was composed of two peristaltic pumps, two oxygenators, a heat exchanger (Xijian Medical), an open bath thermostat (HerryTech), a thermometer, a bubble trap (Xijian Medical), and a chamber. For the NMP group (*n *= 6), the perfusate was maintained at 37℃ and perfused through the portal vein and the hepatic artery at a speed of 8 ml/min and 4 ml/min, respectively, for 4 h. The perfusate consisted of 20 ml rat blood, 50 ml William's E Medium (Sigma), 250 U heparin, 20,000 U penicillin, 2 mg hydrocortisone, 0.4 U insulin, and 0.0292 g glutamine (haematocrit 10–14), with the pO_2,_ maintained at 200–300 mmHg. In addition, the CIRP competitive inhibitor (C23, 300 ng/ml, Bioyears) was added to the perfusate of the HOPE group. In the reperfusion model (*n *= 5), the livers of these four groups (CS I/R, HOPE I/R, HOPE + C23 I/R, and NMP I/R) were perfused using the NMP system for 2 h. Bile produced by the livers was also collected.

### 2.4 Cell culture

Rat Kupffer cells were isolated by density gradient centrifugation using a percoll gradient, as described previously.^15^ The cells were cultured in Dulbecco's Modified Eagle's Medium (DMEM, Gibco) supplemented with 10% foetal bovine serum (FBS, Gibco) and 1% penicillin/ streptomycin (HyClone). After adherence, the Kupffer cells were cultured for 4 h in two conditions: normal temperature (37℃) and normoxia (normal group, *n* = 3), and hypothermia (4℃) and hypoxia (1% O_2_) (hypothermia and hypoxia group, *n* = 3).

Human normal hepatocytes (HL‐7702) were cultured in DMEM at 37℃ in 5% CO_2_. Cells were treated with recombinant human CIRP protein (rhCIRP, Cloud‐Clone Crop.) or C23 in vitro for 6 h. Then, the cells were divided into four groups as follows (*n* = 3/group): control, low rhCIRP (100 ng/ml), high rhCIRP group (1000 ng/ml), and high rhCIRP (1000 ng/ml) + C23 (300 ng/ml).

### 2.5 Histological analysis

Liver tissue samples were fixed in 4% paraformaldehyde, embedded in paraffin and cut into 5 μm sections for haematoxylin and eosin (HE) staining. Liver injury was evaluated according to Suzuki's score by a blinded experimenter. For transmission electron microscopy, 1‐mm^3^ liver tissue samples were fixed in 2.5% glutaraldehyde, and the samples were prepared using routine procedures. Ultrathin sections were contrasted with lead citrate and uranyl acetate and visualized using an HT7800 transmission electron microscope (Hitachi).

### 2.6 Enzyme‐linked immunosorbent assays (ELISA)

Cold‐inducible RNA‐binding protein (SEG886Hu and SEG886Ra, Cloud‐Clone Corp.), mitochondrial transcription factor A (TFAM; SEH050Hu, Cloud‐Clone Corp.), interleukin 6 (IL‐6; E‐EL‐H0109c and E‐EL‐R0015c, Elabscience), interleukin 1 beta (IL‐1β; E‐EL‐R0012c, Elabscience), tumour necrosis factor‐alpha (TNF‐α; E‐EL‐R2856c, Elabscience) and high mobility group protein B1 (HMGB1; E‐EL‐R0505c, Elabscience) ELISA kits were used to detect these molecules in the UW solution, human serum and rat serum.

### 2.7 Measurement of liver function and ICG metabolism

The serum levels of alanine aminotransferase (ALT) and aspartate aminotransferase (AST) were detected using a serum ALT assay kit (C009‐2, NanJing JianCheng Bioengineering Institute) and a serum AST assay kit (C010‐2, NanJing JianCheng Bioengineering Institute). Each DCD liver was weighed after collection. During reperfusion, the bile produced by each liver in the first hour was collected. The bile production was calculated by dividing the bile volume by the liver weight. Indocyanine green (ICG) solution was serially diluted and the OD values at 785 nm were obtained to generate an ICG standard curve. For ICG metabolism analysis, 0.5 g ICG was added to the perfusate at the beginning of the second hour of reperfusion. The bile generated during the second hour was collected and the bile ICG content was calculated using the standard curve. The ICG metabolism was equal to the bile ICG content divided by the liver weight.

### 2.8 ATP content and measurement of oxidative stress

The ATP content in DCD livers after preservation was detected using an ATP assay kit (S0026, Beyotime Biotechnology, *n* = 6). Oxidative stress in the liver after reperfusion was analysed using a malonaldehyde (MDA) assay kit (A003‐1, NanJing JianCheng Bioengineering Institute) using liver tissue homogenate (*n* = 5).

### 2.9 TUNEL, DHE, MitoTracker Red fluorescence staining and apoptosis assays

To detect apoptosis after warm ischaemia (*n *= 6) and reperfusion (*n* = 5), TUNEL staining was performed using a DeadEnd Fluorometric TUNEL System (Promega). The cells were counterstained with DAPI. TUNEL staining was quantified by counting the cells from 5 images per sample. The same blinded experimenter counted all the images. MitoTracker Red CMXRos dye (M7512, Thermo Fisher Scientific) and Dihydroethidium (DHE) dye (D7008, Sigma) were used to detect mitochondria and ROS in cells according to the instructions of the manufacturer. The quantification of fluorescence intensity was performed using ImageJ software. Apoptotic HL‐7702 cells treated with rhCIRP or C23 were assayed using an apoptosis detection kit (A211‐02, Vazyme) and analysed by flow cytometry using a cytoFLEX instrument (Beckman Coulter). The apoptotic cell percentage was calculated using early and late apoptotic cells.

### 2.10 Immunohistochemistry

Immunohistochemical staining of liver sections was performed using paraffin‐embedded sections. The tissues were stained using antibodies against CIRP (10209–2‐AP, Proteintech), CD68 (ab125212, Abcam), vWF (ab216566, Abcam) and MPO (22225–1‐AP, Proteintech).

### 2.11 Western blot

An equal amount of protein from each group was fractionated by 8%–15% sodium dodecyl sulphate‐polyacrylamide gel electrophoresis (SDS‐PAGE). The proteins were transferred onto a PVDF membrane (Millipore, Germany). The membranes were incubated with primary antibody overnight at 4℃, followed by horseradish peroxidase (HRP)‐conjugated secondary antibodies. The antibodies used in this study were anti‐CIRP (Proteintech, 1:500), anti‐caspase‐3 (9662, Cell Signalling Technology, 1:1000), anti‐cleaved caspase‐3 (9661, Cell Signalling Technology, 1:1000), anti‐Fis‐1 (GTX111010, GeneTex, 1:250), anti‐Drp‐1 (ab184247, Abcam, 1:1000), anti‐UCP2 (uncoupling protein 2, 89326, Cell Signalling Technology, 1:1000), anti‐TFAM (22586–1‐AP, Proteintech, 1:200), anti‐TLR‐4 (19811–1‐AP, Proteintech, 1:500), anti‐gp91^phox^ (19013–1‐AP, Proteintech, 1:1000), anti‐p47^phox^ (YT3520, Immunoway, 1:1000) and anti‐β‐actin (4967, Cell Signalling Technology, 1:1000). Proteins were detected using Clarity Western ECL substrate (Bio‐Rad Laboratories) and viewed using a Universal Hood III imaging system (Bio‐Rad).

### 2.12 Statistical analysis

The CIRP and TFAM levels in UW solution and serum are shown as the mean ± SEM. Other data are expressed as the mean ± SD. Pearson correlation analysis was performed to assess relationships between CIRP in human serum 2 h after reperfusion and IL‐6, ALT and AST. Student's *t*‐tests were used to analyse differences between groups. *p *< 0.05 was considered statistically significant. Statistical analysis was performed using SPSS software, version 25 (IBM Corp.).

## 3 RESULTS

### 3.1 CIRP expression in human DCD livers increases during cold storage

**FIGURE 1:**
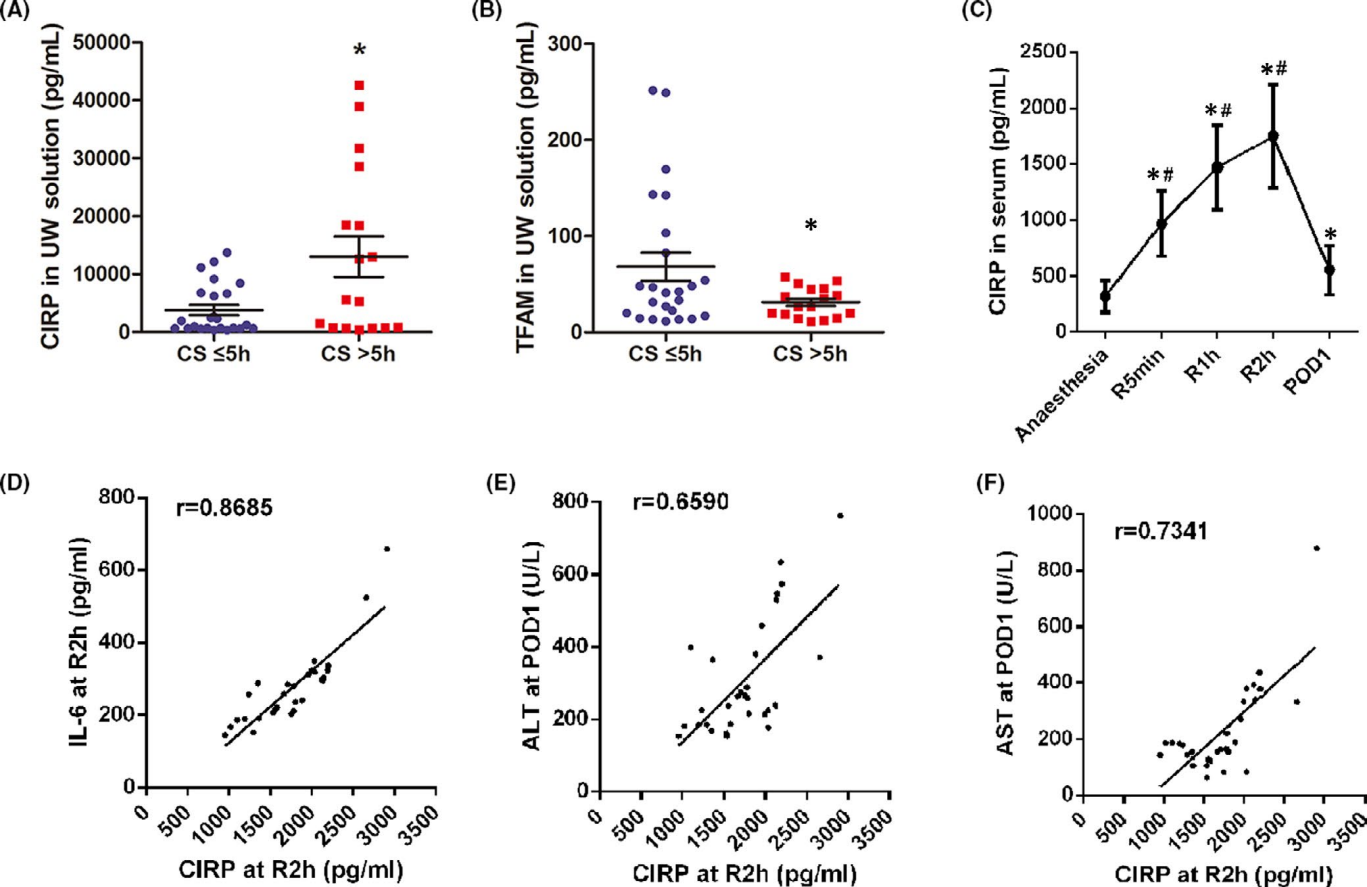
CIRP expression increases in human DCD livers during cold storage. (A) A total of 41 UW specimens were collected after cold storage. CIRP expression in the CS>5 h group was 2.4 times higher than that in the CS≤5 h group. Data are shown as mean ± SEM, *p < 0.05. (B) The CS>5 h group had lower TFAM expression compared to the CS≤5 h group. Data represent mean ± SEM, *p < 0.05. (C) Serum specimens from 30 patients were analysed at five time points. Serum CIRP levels reached the peak at 2 h after reperfusion (R2h) and returned to preoperative levels 1 day after the operation (POD1). Data are shown as mean ± SEM, *p versus the anaesthesia group, ^#^ *p* versus the POD1 group. (D) The expression of CIRP and IL‐6 at R2h are positively correlated (*r* = 0.8685, *p *< 0.001). (E) The expression of CIRP at R2h was positively correlated with ALT at POD1 (*r* = 0.6590, *p *< 0.001). (F) The expression of CIRP at R2h was positively correlated with AST at POD1 (*r* = 0.7341, *p *< 0.001)

According to the cold storage time, 41 UW specimens were divided into two groups: the CS ≤ 5 h group (*n* = 24) and the CS > 5 h group (*n* = 17). CIRP expression in the CS > 5 h group was 2.4 times higher than that in the CS ≤ 5 h group (13022 ± 3513 vs. 3826 ± 873 pg/ml, *p *= 0.021, Figure 1A). In the CS > 5 h group, the specimens with longer storage time had a higher concentration of CIRP. Conversely, the expression of TFAM, an essential protein for mitochondrial biogenesis, was lower in the CS >5 h group (68.4 ± 14.6 vs. 31.3 ± 3.7 pg/ml, *p *= 0.020, Figure 1B). After reperfusion, CIRP was released from the DCD donor liver. The CIRP serum level reached its peak at 2 h after reperfusion (R2h) and was 4.5 times higher than that before anaesthesia (1750 ± 460 vs. 318 ± 144 pg/ml, *p *< 0.001). One day after the operation (POD1), the CIRP level was significantly decreased to levels near preoperative levels (Figure 1C). In addition, CIRP and IL‐6 expression at R2h were positively correlated (*r* = 0.8685, *p *< 0.001, Figure 1D). Further, CIRP expression at R2h was positively correlated with ALT and AST at POD1 (*r* = 0.6590 and 0.7341, respectively, *p *< 0.001, Figure 1E, F).

### 3.2 Warm ischaemia increases CIRP secretion in the liver and mediates mitochondrial dysfunction

**FIGURE 2:**
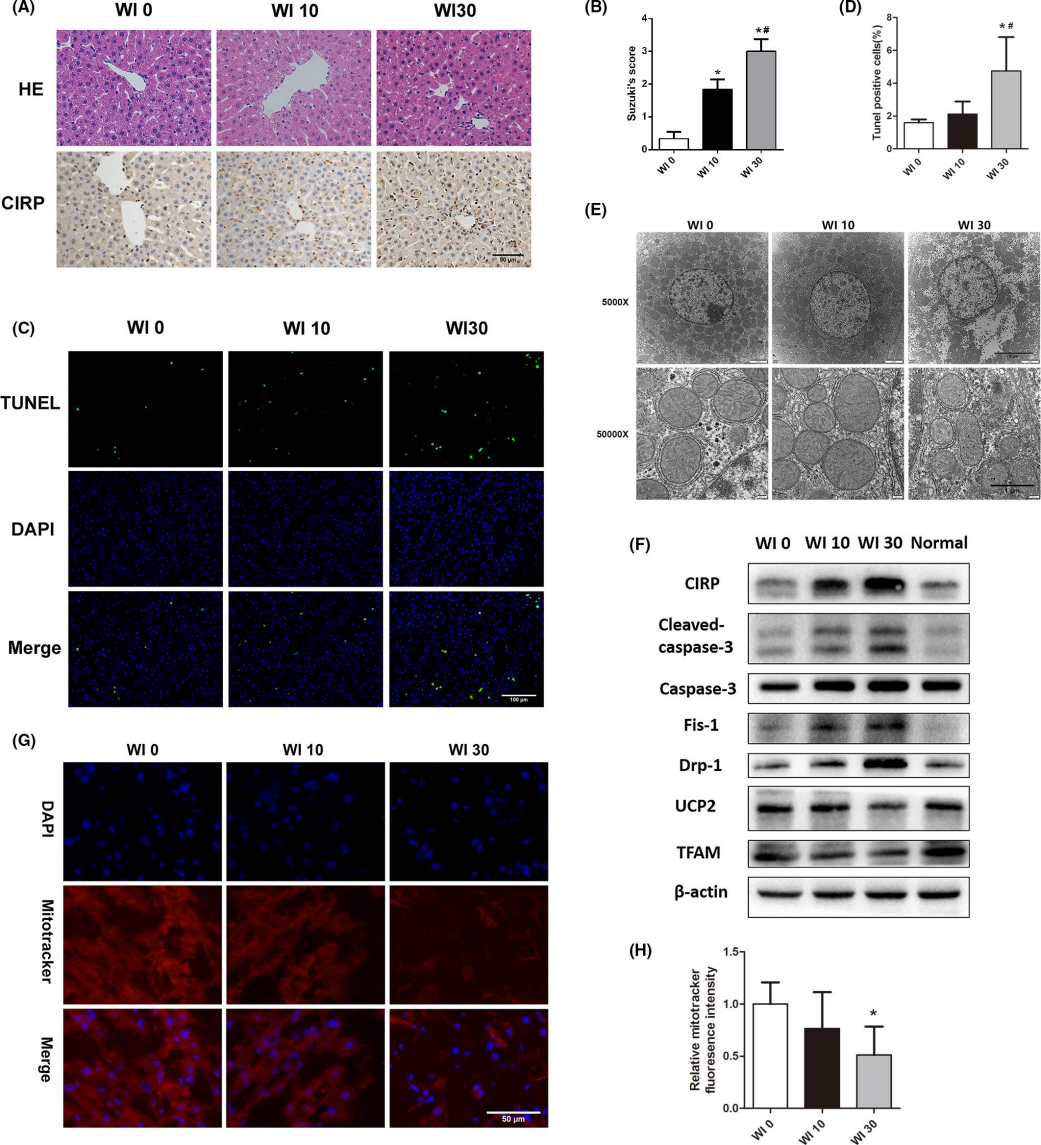
Warm ischaemia increases CIRP secretion in the liver and mediates mitochondrial dysfunction. (A) HE staining and CIRP immunohistochemistry of DCD livers with different warm ischaemia times (*n* = 6, magnification 200×). (B) According to Suzuki's score, the liver injury in the WI 30 group was more severe than that in the other two groups. (C) TUNEL staining of DCD liver with different warm ischaemia times (magnification 200×). (D) The number of apoptotic cells in the WI 30 group was significantly higher than in the WI 0 and WI 10 groups. (E) Transmission electron microscopy of liver tissues. In the WI 30 group, the density of mitochondria was significantly decreased, and the outer membrane of mitochondria was broken and the mitochondrial cristae disintegrated. (F) Western blot analysis of CIRP and mitochondrial proteins. (G and H) MitoTrachker Red fluorescence staining and its fluorescence intensity of DCD liver after warm ischaemia. *p < 0.05 versus the WI 0 group, ^#^ *p* < 0.05 versus the WI 10 group. Scale bars: 50 μm (A and G), 100 μm (C), 5 μm (top, E) and 1 μm (bottom, E)

The rat DCD model was used to analyse the effect of warm ischaemia time on CIRP secretion. HE staining showed that some hepatocytes in the WI 30 group presented vacuolar degeneration (Figure 2A). The Suzuki score of this group was significantly higher than that of the WI 0 and WI 10 groups (*p *< 0.05, Figure 2B). With prolonged warm ischaemia time, CIRP expression in Kupffer cells increased (Figure 2A). The apoptosis rate in the WI 30 group was significantly higher than that in the other two groups (4.7% ± 2.1% vs. 1.6% ± 0.2%, *p *= 0.013; 4.7% ± 2.1% vs. 2.1% ± 0.8%, *p*=0.025, Figure 2C, D). TEM showed that the number of mitochondria in the WI 30 group decreased significantly, and nuclear oedema with abnormal morphology. At high magnification, the outer membrane of mitochondria was broken and the mitochondrial cristae disintegrated in WI 30 group (Figure 2E). Prolonged warm ischaemia time increased CIRP and cleaved caspase‐3 expression and promoted mitochondrial fission by upregulating Fis‐1 and Drp‐1. At the same time, sustained ischaemia reduced TFAM and UCP2, which may have caused impaired mitochondrial biogenesis and mitochondrial dysfunction (Figure 2F). MitoTracker fluorescence intensity was also markedly decreased in WI 30 group (Figure 2G, H).

### 3.3 Cold storage promotes CIRP secretion by Kupffer cells

**FIGURE 3:**
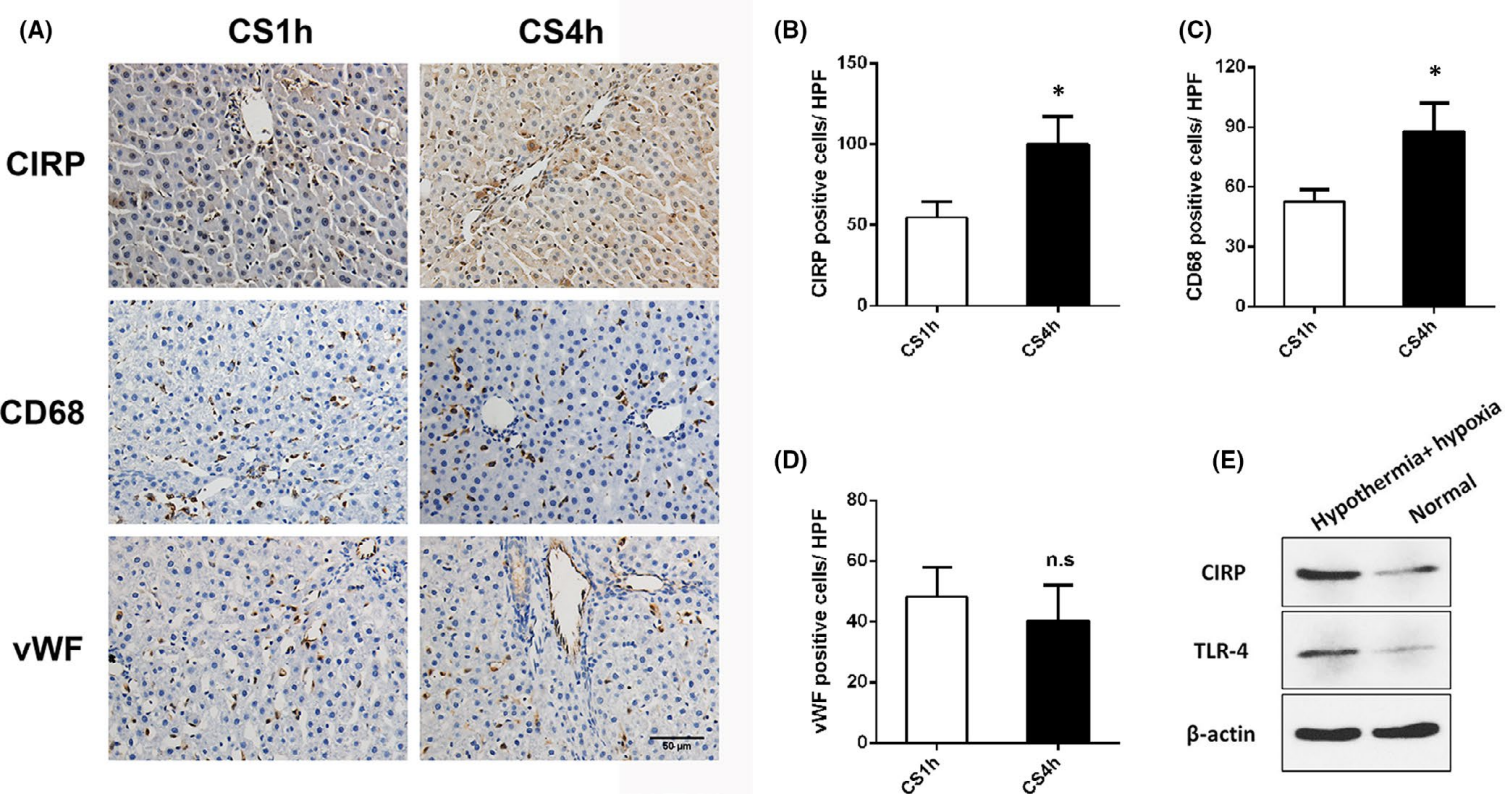
Cold storage promotes CIRP secretion in Kupffer cells. (A) Immunohistochemical staining of CIRP, CD68, and vWF in DCD livers, which were in cold storage for 1 h and 4 h (magnification 200×). (B) The number of CIRP‐positive cells in the CS4h group was significantly higher than in the CS1h group (*n* = 6). (C) The number of CD68‐positive cells in the CS4h group was higher than in the CS1h group. (D) There was no difference between the two groups for the number of vWF‐positive cells (*p *= 0.277). (E) Kupffer cells cultured in a hypothermic/hypoxia environment had upregulated expression of CIRP and TLR‐4 compared to cells cultured in a normal environment. *p < 0.05 versus CS1h. Scale bars: 50 μm (A)

Rat DCD liver was stored in the cold for 1 (CS1h) or 4 (CS4h) hours. The number of CIRP‐positive cells in the CS4h group was significantly higher than that in the CS1h group. CIRP was mainly expressed in non‐parenchymal cells (Kupffer cells, *p *= 0.002, Figure 3A, B). The number of CD68‐positive cells in the CS4h group was higher than that in the CS1h group (*p *= 0.003, Figure 3C). However, there was no difference between the two groups for the number of vWF‐positive cells (*p *= 0.277, Figure 3D). This suggests that cold storage may cause more damage to Kupffer cells than to vascular endothelial cells. Furthermore, rat primary Kupffer cells were cultured in hypothermic/hypoxic and normal environments. The results indicated that CIRP and TLR‐4 expression were upregulated in Kupffer cells cultured in a hypothermic/hypoxic environment (Figure 3E).

### 3.4 NMP reduces CIRP secretion and mitochondrial dysfunction in DCD liver

**FIGURE 4:**
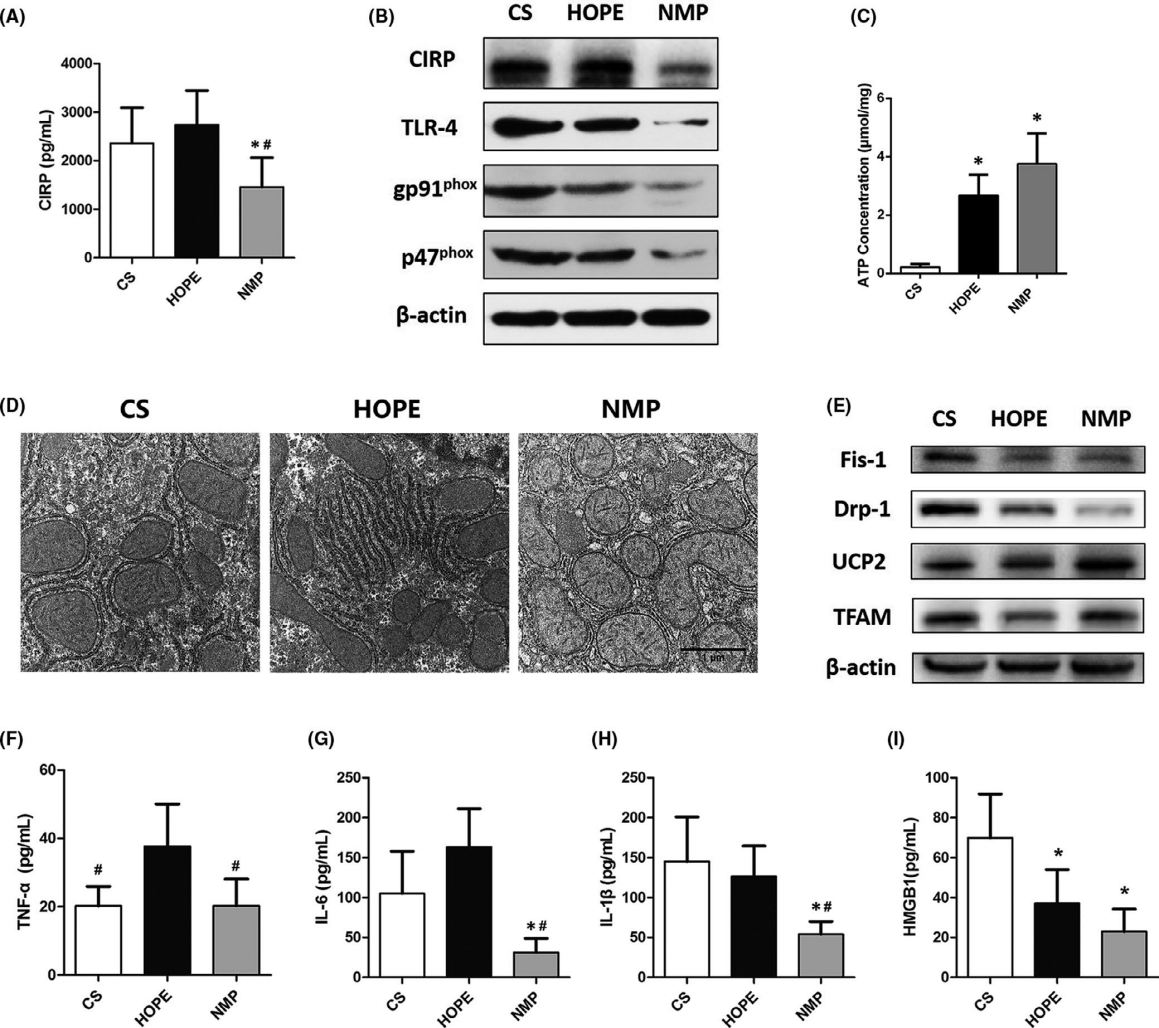
NMP reduces CIRP secretion and mitochondrial dysfunction in the DCD liver. The DCD livers were preserved for 4 h in vitro using CS, HOPE and NMP, respectively (*n* = 6). (A) The expression of CIRP in the perfusate of the three different groups. (B) The expression of CIRP, TLR‐4, and NADPH oxidase in liver tissues. (C) ATP content in the livers of the three groups. (D) TEM images of liver tissues. Liver tissues in the NMP group had a high mitochondrial density, clear mitochondrial membrane and cristae, and without expanded endoplasmic reticulum (magnification 20000×). (E) Western blot analysis of mitochondrial proteins. The expression of inflammatory factors including TNF‐α (F), IL‐6 (G), IL‐1β (H), and HMGB1 (I) in the perfusates. *p < 0.05 versus the CS group, ^#^ *p* < 0.05 versus the HOPE group. Scale bars: 1 μm (D)

Three different strategies were used to preserve DCD livers in vitro for 4 h. CIRP expression in the perfusate of the NMP group was significantly lower than that in the CS and HOPE groups (NMP vs. CS *p* = 0.044, NMP vs. HOPE *p* = 0.007, Figure 4A). Further, CIRP, TLR‐4, and NADPH oxidase in the liver tissue of the NMP group were lower than those in the other two groups (Figure 4B). In addition, we observed significantly decreased ATP content in the liver tissues of the CS group (CS vs. HOPE *p* = 0.025, CS vs. NMP *p* = 0.027, Figure 4C). TEM showed that the liver tissues in the NMP group had a higher density of mitochondria and a more defined mitochondrial membrane and cristae. These cells also had less expanded endoplasmic reticulum compared to that in the other two groups (Figure 4D). UCP2 plays an important role in the regulation of mitochondrial function. UCP2 and TFAM expression were increased in the NMP group (Figure 4E). Moreover, NMP also inhibited the expression of mitochondrial fission proteins Fis‐1 and Drp‐1. The expression of inflammatory factors, including TNF‐α, IL‐6, IL‐1β, and HMGB1 in the perfusate of the NMP group was significantly lower than those in the CS and HOPE groups (Figure 4F–I).

### 3.5 NMP attenuates CIRP‐mediated ischaemia‐reperfusion injury in DCD livers

**FIGURE 5:**
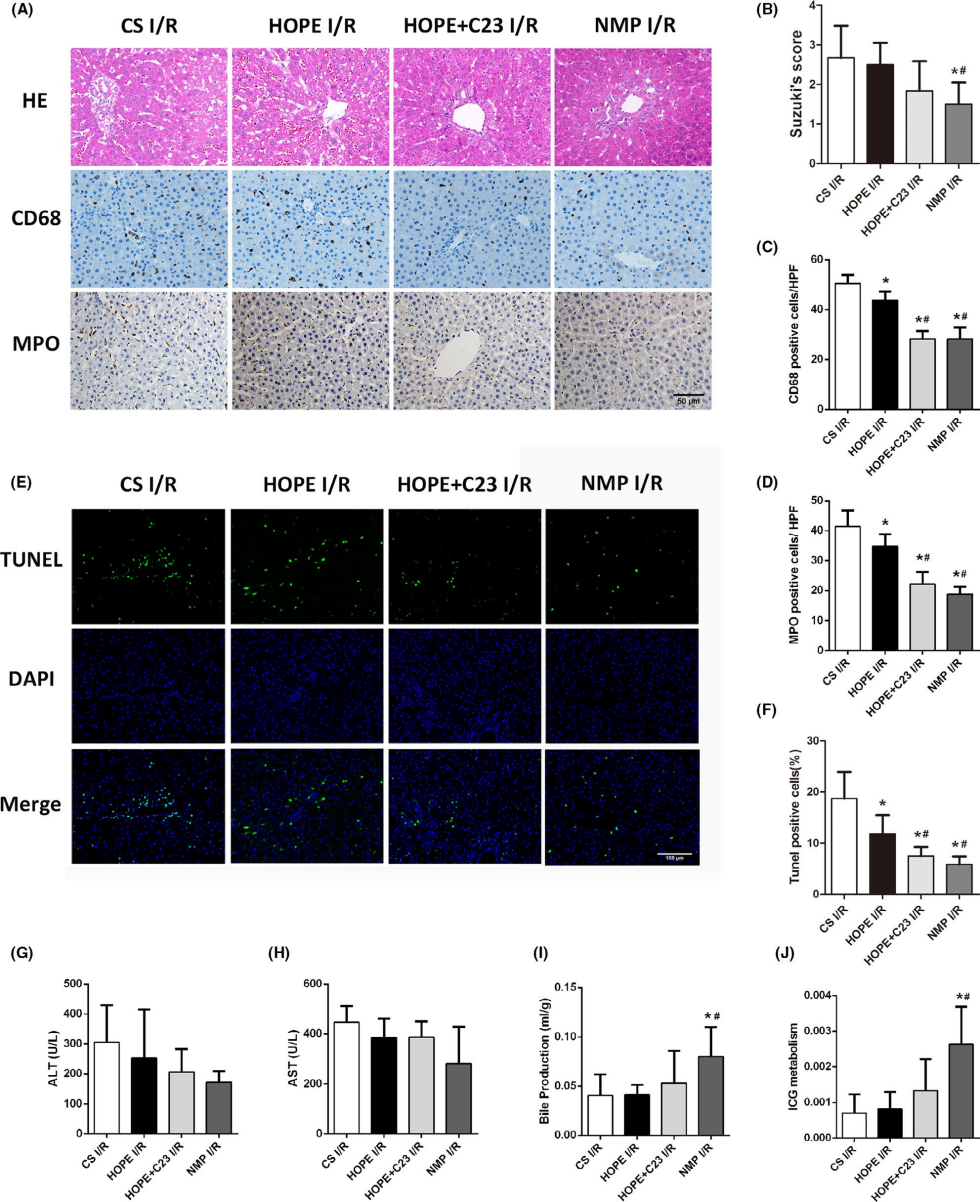
NMP attenuates ischaemia‐reperfusion injury in DCD livers. The competitive CIRP inhibitor C23 (300 ng/ml) was mixed into the perfusate of the HOPE group. The livers of the four groups were re‐perfused using the NMP system for 2 h (*n* = 5). (A) HE staining, CD68, and MPO immunohistochemistry of representative liver sections (magnification 200×). (B) Liver injury in the CS I/R and HOPE I/R groups was more severe than that in the NMP I/R group. (C) The number of CD68‐positive cells and (D) MPO‐positive cells in each group. (E) TUNEL staining. (F) The number of TUNEL‐positive cells in each group. (G) ALT levels in the perfusate of the four groups. (H) AST levels in the perfusate of the four groups. (I) Bile production in the first hour of reperfusion in each group. (J) Liver ICG metabolism in each group during the second hour of reperfusion. ^*^ *p *< 0.05 versus the CS I/R group, ^#^ *p *< 0.05 versus the HOPE I/R group. Scale bars: 50 μm (A) and 100 μm (E)

The DCD livers of the four groups were re‐perfused with the NMP system for 2 h. Suzuki's score showed that the injury to the liver tissues in the CS I/R and HOPE I/R groups were more serious than that in the NMP I/R group (*p *< 0.05, Figure 5B). To determine the alterations in Kupffer cell activation and neutrophil infiltration, immunohistochemical staining of CD68 and MPO were performed (Figure 5A). The number of CD68‐ and MPO‐positive cells in the NMP I/R and HOPE + C23 I/R groups was significantly less than that in the other two groups (*p *< 0.05, Figure 5C, D). In addition, TUNEL staining indicated that the number of apoptotic cells significantly decreased in the NMP I/R and HOPE+C23 I/R groups (*p *< 0.05, Figure 5E, F). For liver function after reperfusion, bile production in the NMP I/R group was twice that in the CS I/R and HOPE I/R groups, and had three times higher ICG metabolism than the CS I/R and HOPE I/R groups (*p *< 0.05, Figure 5I, J). However, there was no difference in the ALT and AST levels between the four groups, which may be related to the short reperfusion time (Figure 5G, H).

**FIGURE 6:**
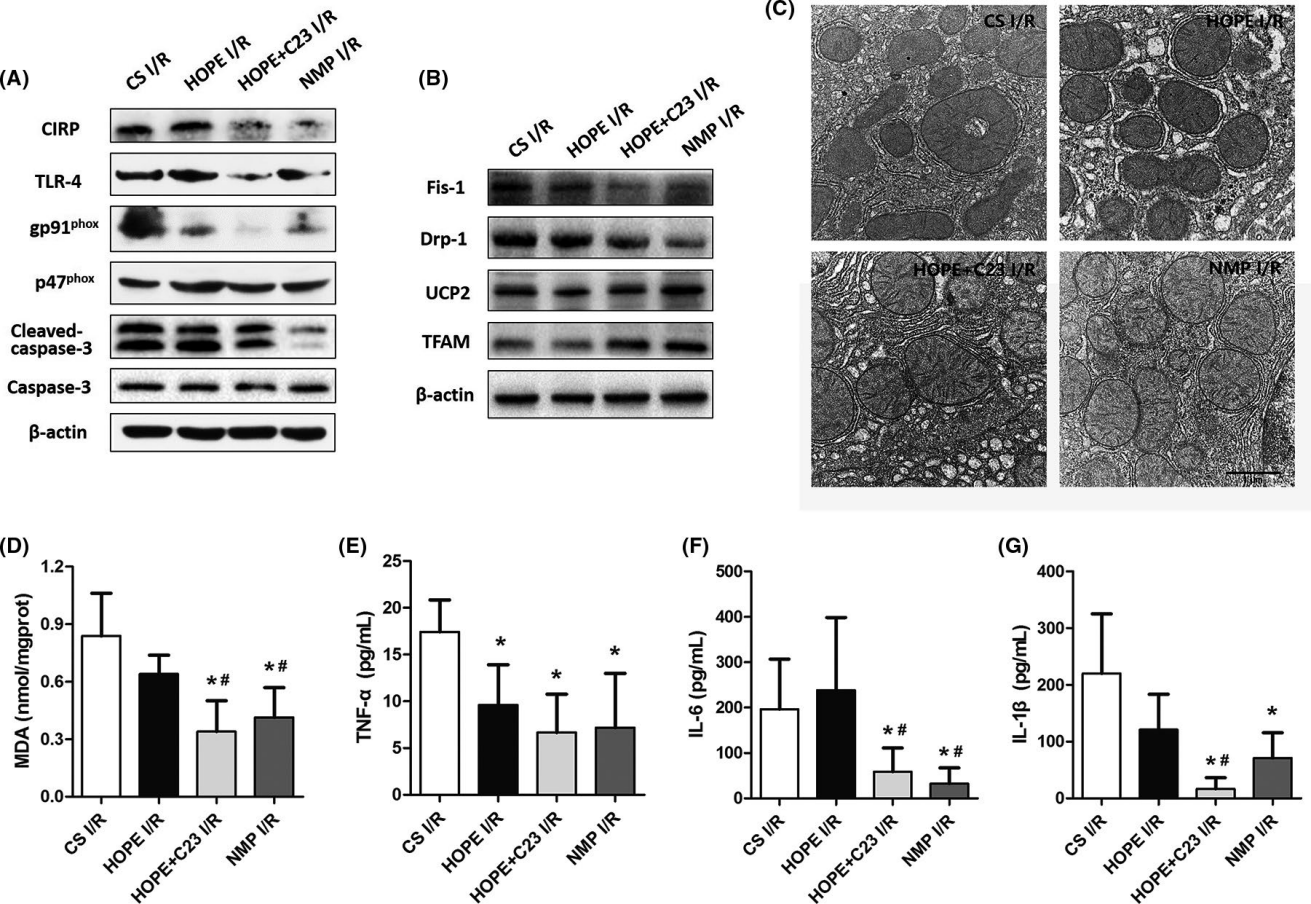
NMP attenuates oxidative stress and mitochondrial dysfunction in the liver after reperfusion. (A) The livers in the NMP I/R and HOPE + C23 I/R groups had low expression of CIRP, TLR‐4, and NADPH oxidase. (B) Western blot analysis of mitochondrial proteins. (C) TEM images of liver tissues in the four groups (magnification 20000×). (D) The livers in the NMP I/R and HOPE +C23 I/R groups had significantly lower MDA levels compared to the CS I/R and HOPE I/R groups. The expression of inflammatory factors, including TNF‐α (E), IL‐6 (F), and IL‐1β (G), in the perfusates, were analysed. *p < 0.05 versus the CS I/R group, ^#^ *p *< 0.05 versus the HOPE I/R group. Scale bars: 1 μm (C)

Oxidative stress is a crucial factor in the process of hepatic IRI. The MDA level in the NMP I/R group was significantly lower than that in CS I/R and HOPE I/R groups (*p *< 0.05, Figure 6D). Moreover, the livers in the NMP I/R group had low CIRP, TLR‐4, and NADPH oxidase expression. The decreased expression of cleaved caspase‐3 indicated a low apoptosis level in the liver, which was consistent with the TUNEL staining results (Figure 6A). NMP also attenuated mitochondrial dysfunction (Figure 6B). C23 administration decreased MDA in the HOPE I/R group by 46.9%, inhibited CIRP and NADPH oxidase expression, reduced mitochondrial fission and promoted biogenesis (Figure 6A, B and D). Compared with the CS I/R group, the mitochondrial membrane and cristae in the NMP I/R and HOPE + C23 I/R groups were much clearer and less damaged (Figure 6C). The expression of inflammatory factors in the NMP I/R group was significantly lower than in the CS I/R group (*p *< 0.05, Figure 6E–G). C23 administration reduced the expression of IL‐6 and IL‐1β by 75.4% and 86.1%, respectively, in the HOPE I/R group (*p *< 0.05, Figure 6F, G).

### 3.6 CIRP mediates oxidative stress and mitochondrial dysfunction in hepatocytes

**FIGURE 7:**
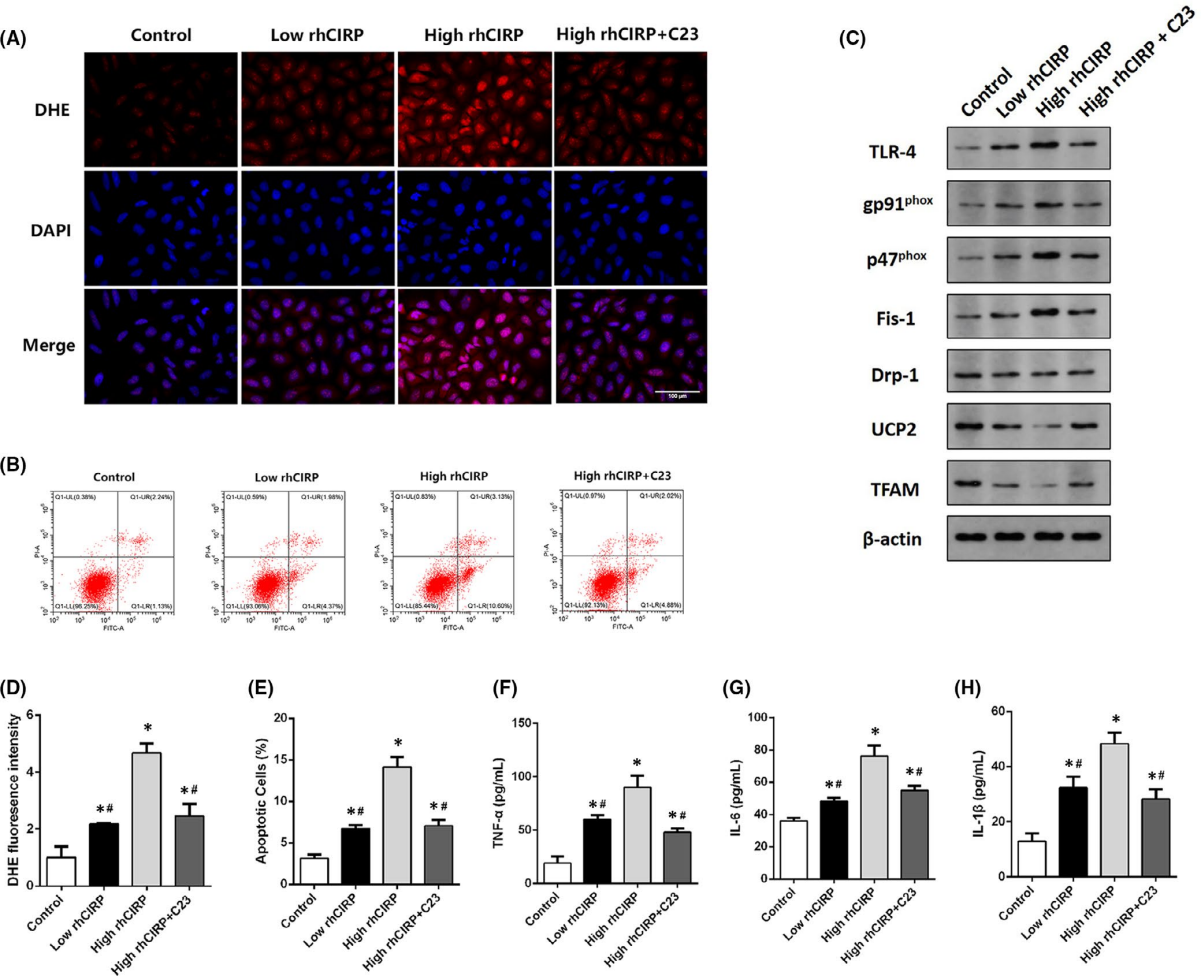
CIRP‐mediated oxidative stress and mitochondrial dysfunction in hepatocytes. HL‐7702 cells were treated with 100 ng/ml rhCIRP, 1000 ng/ml rhCIRP, or 1000 ng/ml rhCIRP +300 ng/ml C23 for 6 h (*n* = 3). (A) DHE fluorescence in the four HL‐7702 cell groups (magnification 400×). (B) Flow cytometry analysis of HL‐7702 cell apoptosis in the four groups. (C) Western blot analysis of NADPH oxidase and mitochondrial proteins. (D) DHE fluorescence of HL‐7702 cells in the four groups. (E) Apoptosis of HL‐7702 cells. The expression of inflammatory factors, including TNF‐α (F), IL‐6 (G), and IL‐1β (H) in the medium. *p < 0.05 versus the control group, ^#^ *p *< 0.05 versus the high rhCIRP group. Scale bars: 100 μm (A)

To verify the effect of CIRP on hepatocytes, HL‐7702 human hepatocytes were treated with rhCIRP and C23. DHE fluorescence intensity was increased after rhCIRP administration. C23 significantly reversed ROS generation and reduced CIRP‐induced oxidative stress (Figure 7A, D). 1000 ng/ml rhCIRP significantly increased the apoptosis rate, which was 4.5 times higher than in the control group and 2.1 times higher than in the low rhCIRP group (*p* < 0.05, Figure 7B, E). Moreover, high rhCIRP concentrations upregulated the expression of NADPH oxidase and promoted mitochondrial fission. rhCIRP also affected mitochondrial biogenesis and energy metabolism by downregulating the expression of TFAM and UCP2 (Figure 7C). Additionally, rhCIRP significantly promoted the secretion of inflammatory factors in hepatocytes (Figure 7F–H). However, C23 alleviated CIRP‐mediated oxidative stress and mitochondrial dysfunction (Figure 7C, D), reduced apoptosis (Figure 7E), and inhibited the secretion of inflammatory factors (Figure 7F–H).

## 4 DISCUSSION

In the present study, we found that human DCD livers secrete a large amount of CIRP after transplantation, which is related to postoperative IL‐6 levels and liver function. In our rat model, CIRP expression was upregulated during warm ischaemia and with increased CS duration. Compared with CS methods, NMP attenuated the IRI of DCD liver by inhibiting CIRP‐mediated oxidative stress and mitochondrial fission.

Static cold storage is a standard strategy for liver preservation in the clinic. However, complications after transplantation are related to the cold ischaemia time of grafts.^16^, ^17^ A lot of evidence have demonstrated that IRI of grafts is associated with organ inflammation characterized by the release of inflammatory mediators, upregulation of adhesion molecules and increased immune cell infiltration.^3^, ^10^, ^18^ Jassem et al.^3^ performed a microarray analysis of liver tissue and found that recipients receiving CS grafts had a higher level of immune‐related gene expression, especially proinflammatory cytokines, compared to the NMP group. In our study, CIRP was secreted during warm ischaemia and cold storage, and CIRP expression increased with prolonged cold storage time. Clinical data also indicated that secreted CIRP is released into the blood soon after transplantation, and is closely related to systemic inflammatory responses and liver function on the first day after surgery.

Reactive oxygen species production is initiated by the generation of superoxide anions by NADPH oxidase.^19^ NADPH oxidase is composed of four cytosolic proteins (P47^phox^, p67^phox^, p40^phox^, and Rac2) and two transmembrane proteins (gp91^phox^ and p22^phox^). gp91^phox^ is the catalytic subunit of NADPH oxidase.^20^ Previous studies demonstrated that NADPH oxidase could be activated in macrophages^14^ and lung vascular endothelial cells^21^ by TLR‐4/MyD88 signalling, which causes excessive ROS production. In our study, the expression of gp91^phox^ and P47^phox^ in the CS group was higher than that in the NMP group after cold preservation and reperfusion, which was consistent with MDA levels. In addition, high rhCIRP concentrations upregulated gp91^phox^ and P47^phox^ expression in HL‐7702 cells, increased ROS, promoted the secretion of inflammatory factors, and induced apoptosis. However, inhibiting CIRP expression effectively downregulated oxidative stress in tissues and cells, thereby reducing IRI.

Mitochondrial fusion and fission, and mitochondrial biogenesis and degradation are two opposed processes that regulate mitochondrial dynamics.^22^, ^23^ In cold storage, hypoxia and energy interruption may result in decreased intracellular ATP, which increases mitochondrial fission to maintain mitochondrial quantity.^24^ However, excessive mitochondrial fission leads to mitochondrial fragmentation and triggers apoptosis.^25^ Mitochondrial fission is mediated by several proteins, but Drp‐1 is the most central.^26^ Zhang et al.^27^ found that inhibiting the translocation and activation of Drp‐1 could prevent hepatocyte apoptosis in liver IRI. In addition, sustained hypoxia and ischaemia also reduce TFAM, leading to impaired mitochondrial biogenesis.^28^ Excessive ROS accumulation can cause mitochondrial dysfunction, and disturbing mitochondrial dynamics increases mitochondrial ROS production. UCPs are mitochondrial anion carrier proteins that play important roles in minimizing ROS emission from the electron transport chain.^29^ Bi et al.^12^ showed that increased UCP2 expression might quench oxidative stress in hepatic IRI. In the current study, TFAM expression in the CS ≤ 5 h group was 2.2 times higher than that in the CS > 5 h group. The result suggests that mitochondrial biogenesis in the DCD liver decreases with prolonged preservation time. Moreover, there were higher Fis‐1 and Drp‐1 expression and lower TFAM and UCP2 expression in the liver of the CS group after in vitro preservation and reperfusion, compared to expression in the NMP group. These results indicate that NMP maintains liver metabolism, attenuates mitochondrial dysfunction, and inhibits inflammation related to IRI.

Hypothermic oxygenated machine perfusion^30^ and NMP^1^, ^2^, ^31^ are two strategies to reduce liver IRI. Both approaches have achieved exciting results in clinical trials. In this study, we did not intend to compare the effects of these two methods on the IRI of DCD livers. The metabolism level of the whole liver was downregulated in the HOPE group after cold perfusion, and the ATP content in the livers after preservation was significantly higher than that in the CS group. However, CIRP was highly expressed in liver tissue and perfusate of the HOPE group. Oxidative stress, inflammatory responses, and apoptosis in liver tissue decreased significantly after the administration of a CIRP inhibitor. Thus, targeting CIRP may also provide a new therapeutic strategy to attenuate oxidative stress and reduce the IRI of the DCD liver during cold preservation. Additionally, we did not use a rat liver transplantation model. Thus, the effect of CIRP on the survival rate of liver transplantation remains to be determined.

## 5 CONCLUSIONS

Cold‐inducible RNA‐binding protein has attracted increasing attention as a novel DAMP. Our study demonstrates that the DCD liver could secrete a large amount of CIRP after ischaemia and cold preservation. Secreted CIRP may induce NADPH oxidase‐derived ROS, leading to mitochondrial fission, inflammatory responses, and apoptosis. NMP can provide energy support for the DCD liver and inhibit CIRP secretion in the liver, thereby alleviating oxidative stress and IRI. Further experiments need to be performed in a rat liver transplantation model.

## CONFLICT OF INTEREST

The authors have declared that no competing interests exist.

## AUTHOR CONTRIBUTIONS

**Wenyan Liu:** Data curation (lead); Formal analysis (equal); Methodology (lead). **Yang Fan:** Methodology (equal). **Hongfan Ding:** Investigation (equal); Methodology (equal). **Dan Han:** Methodology (equal). **Yang Yan:** Supervision (equal). **Rongqian Wu:** Supervision (equal); Writing‐review & editing (equal). **Yi Lv:** Supervision (equal). **Xinglong Zheng:** Data curation (equal); Funding acquisition (lead); Writing‐original draft (lead); Writing‐review & editing (lead).

## Data Availability

The data that support the findings of the present study are available from the corresponding author upon reasonable request.
